# Nicotinamide reduces renal interstitial fibrosis by suppressing tubular injury and inflammation

**DOI:** 10.1111/jcmm.14285

**Published:** 2019-04-16

**Authors:** Meiling Zheng, Juan Cai, Zhiwen Liu, Shaoqun Shu, Ying Wang, Chengyuan Tang, Zheng Dong

**Affiliations:** ^1^ Department of Nephrology, The Key Laboratory of Kidney Disease and Blood Purification of Hunan Province Second Xiangya Hospital at Central South University Changsha China; ^2^ The State Key Laboratory of Medical Genetics, School of Life Sciences Central South University Changsha China

**Keywords:** apoptosis, inflammation, NAD^+^, renal fibrosis, tubular atrophy

## Abstract

Renal interstitial fibrosis is a common pathological feature in progressive kidney diseases currently lacking effective treatment. Nicotinamide (NAM), a member of water‐soluble vitamin B family, was recently suggested to have a therapeutic potential for acute kidney injury (AKI) in mice and humans. The effect of NAM on chronic kidney pathologies, including renal fibrosis, is unknown. Here we have tested the effects of NAM on renal interstitial fibrosis using in vivo and in vitro models. In vivo, unilateral urethral obstruction (UUO) induced renal interstitial fibrosis as indicated Masson trichrome staining and expression of pro‐fibrotic proteins, which was inhibited by NAM. In UUO, NAM suppressed tubular atrophy and apoptosis. In addition, NAM suppressed UUO‐associated T cell and macrophage infiltration and induction of pro‐inflammatory cytokines, such as TNF‐α and IL‐1β. In cultured mouse proximal tubule cells, NAM blocked TGF–β‐induced expression of fibrotic proteins, while it marginally suppressed the morphological changes induced by TGF‐β. NAM also suppressed the expression of pro‐inflammatory cytokines (eg MCP‐1 and IL‐1β) during TGF‐β treatment of these cells. Collectively, the results demonstrate an anti‐fibrotic effect of NAM in kidneys, which may involve the suppression of tubular injury and inflammation.

## 1 INTRODUCTION

Renal tubulointerstitial fibrosis is a common pathological feature of progressive kidney diseases^1^, ^2^ including chronic kidney disease CKD, a growing global health problem.^6^ Tubular atrophy, deposition of extracellular matrix (ECM), and myofibroblast expansion are the notable characteristics of renal fibrosis. The pathogenesis of renal fibrosis is very complex and still incompletely understood, but it is generally accepted that it involves tubular pathologies, inflammation and infiltration of inflammatory cells, activation and expansion of fibroblasts, and dropout of microvasculature.^1^, ^2^ There is an urgent need to identify anti‐fibrotic medicines that may offer effective therapies for CKD and related fibrotic diseases.

Nicotinamide (NAM) is a member of the water‐soluble vitamin B family, which can be produced in vivo or taken from the diet or food, including meat, dairy products, green leafy vegetables, seeds, beans, nuts and grains.^7^ In cells, NAM is the substrate for the synthesis of NAM adenine dinucleotide (NAD) and NAM adenine dinucleotide phosphate (NADP+), critical coenzymes in glycolysis, the citric acid cycle and the electron transport chain of respiration.^8^ NAM has been used as a dietary supplement.^9^ In addition, therapeutic effects of NAM have been reported in a range of diseases from pellagra, sepsis, to type I diabetes and fatty liver.^10^, ^11^ Mechanistically, NAM may modulate fatty acid metabolism, inflammation, oxidative stress, cell proliferation and apoptosis. In kidneys, Tran et al showed that supplementation of NAM may reverse established ischaemic acute kidney injury (AKI).^13^ Remarkably, Poyan et al further demonstrated the therapeutic potential in human AKI patients.^14^ However, the effect of NAM in chronic kidney disease and related renal interstitial fibrosis, has not been reported. In this study, we conducted in vivo and in vitro experiments to examine the effects of NAM on the development of chronic renal pathologies, including renal interstitial fibrosis.

## 2 MATERIALS AND METHODS

### 2.1 Antibodies and reagents

Following primary antibodies were used in this study: anti‐α‐SMA (19245), anti‐cleaved‐caspase3 (9664), anti‐vimentin (3932) and anti‐GAPDH (5174) from Cell Signaling Technology; anti‐Fibronectin (NBP1‐91258) from Novus Biologicals and anti‐F4/80 (GB11027) from Servicebio. Secondary antibodies were purchased from Thermo‐Fisher Scientific. NAM (72340) was purchased by Sigma‐Aldrich, and recombinant human TGF–β1 (GF111) was from EMD Millipore.

### 2.2 Mouse model of unilateral urethral obstruction

Male C57BL/6 mice (8 weeks to 10 weeks) were purchased from Hunan Slack King Experimental Animal Company (Changsha, China). Unilateral ureteral obstruction surgery was performed by the procedure described before.^15^, ^16^ Briefly, mice were anaesthetized with pentobarbital (60 mg/kg) and mouse body temperature was maintained at approximately 36.5°C by a rectal probe of the Homeothermic Blanket Control Unit (507220F, Harvard Apparatus). A small incision was made on the left side of the mouse to expose and separate the ureter. In the middle of left ureter, 2 points were sutured with 4‐0 silk thread. The control mice only exposed the left ureter to separate without ligation. For NAM treatment, mice were intraperitoneally injected with different doses of NAM (200 mg/kg, 400 mg/kg, 800 mg/kg) one hour before unilateral urethral obstruction (UUO) surgery, and then injected daily at the same point in time until the day before being sacrificed. The mice were sacrificed at 14 days after UUO surgery and their left kidneys were harvested for biochemical and morphological examinations.

### 2.3 Cells and TGF‐β treatment

The Boston University mouse proximal tubular cell line (BUMPT) was originally provided by Dr Wilfred Lieberthal (Boston University School of Medicine). The cells were cultured in DMEM medium containing 10% foetal bovine serum. Briefly, BUMPT cells were uniformly plated in a density of 0.3 × 10^6^ cells/dish in a 35mm dish and starved overnight in serum‐free DMEM media. The cells were then incubated with 5 ng/mL TGF‐β for 48 hours in serum‐free DMEM medium, while control cells were maintained in serum‐free medium without TGF‐β. To test the effect of NAM, 25 µmol/L of NAM was added. After 48 hours treatment, cell morphology was monitored and cells were harvested for biochemical analysis.

### 2.4 Apoptosis detection in BUMPT cells and kidney tissues

Apoptosis in BUMPT cells and kidney tissues were determined by two methods. First, apoptosis of cells and kidney tissues were determined by Western blot analysis of cleaved‐caspase3 as recently described.^17^, ^18^ Second, morphological analysis was used to analyse apoptosis in cultured cells. The cells were stained with Hoechst 33342 (Molecular Probes, H1399) and the cells which showed typical apoptotic morphological features, including nuclear condensation and chromatin fragmentation, were counted to determine the percentage of apoptosis. TUNEL staining was performed in kidney tissues according to the protocol of the Cell Death Detection kit (Roche Applied Science, 12156792910) as described in our previous work.^17^, ^18^ For quantification, 10 to 20 fields were selected randomly from each tissue section to count TUNEL‐positive cells/mm^2^.

### 2.5 Histological analysis of kidney tissues

Kidney tissue was fixed in 4% paraformaldehyde solution, embedded in paraffin and sectioned at 4µm. Haemotoxylin and eosin (H&E) staining and Masson trichrome staining were performed according to the standard protocols provided by the manufacturer (Servicebio). The atrophic renal tubules were characterized by thinning of tubular cell body, dilation of the tubule, necrotic debris in the lumen, and significant expansion of the interstitial space. Tubular atrophy score was calculated by using a scoring system based on the percentage of atrophic tubules (0; 1, <25%; 2, 25% to 50%; 3, 50%‐75%; 4, >75%). The type I, type III and type IV collagen fibrils were stained aniline blue by Masson staining. To quantify the fibrotic area, 10 to 20 stained areas (magnification 100×) were randomly selected. ImageProPlus software was then used to assess the ratio of positively (blue) stained area to the entire area (excluding glomerular, small vena cava and blood vessels), which was expressed as the percentage of fibrotic area.

### 2.6 Immunohistochemical analysis of T cells and macrophages

Paraffin‐embedded tissue sections were deparaffinized, rehydrated and placed in 0.1 mol/L sodium citrate (pH 6.0). Then the paraffin sections were heated in an oven for antigen retrieval. These sections were incubated in 3% hydrogen peroxide and blocked in 2% normal goat serum. The sections were then exposed to 1:250 anti‐CD3 or 1:1000 anti‐F4/80 antibodies overnight at 4°C, followed by rewarming at room temperature and exposure to biotinylated goat anti‐rabbit secondary antibody (Beijing Zhongshan Jinqiao Biotechnology, VP‐9000). Negative controls were incubated in antibody diluent. The colour was developed with a DAB kit (Beijing Zhongshan jinqiao Biotechnology, ZLI‐9018). Randomly selected 10‐20 fields from each sections (magnification 100×) were examined to count the cells in interstitium with positive CD3 staining as T cells, and the cells with positive F4/80 staining as macrophages.

### 2.7 Western blot analysis

Cells and kidney tissues were lysed in a lysis buffer containing 2% SDS and protease inhibitor cocktail (Sigma‐Aldrich, P8340). The lysates were assayed for protein concentration by the BCA Protein Assay Kit (Thermo Scientific, 23225) and then loaded for reduced SDS‐gel electrophoresis by standard procedures. The proteins were then transferred onto polyvinylidene difluoride membranes. These membranes were incubated with blocking buffer containing 5% bovine serum albumin and then exposed to specific primary antibodies overnight at 4°C. Finally, the membranes were incubated with secondary antibodies to reveal signal with the ECL kit (EMD Millipore, WBKLS0500).

### 2.8 Real‐time RT‐PCR

For quantitative analysis of gene expression, total RNA was isolated using the Trizol kit (12183555, Invitrogen,). RNA concentration was measured by NanoDrop2000 and cDNA was synthesized by using the Takara kit (RR047A, Takara). RT‐PCR was performed according to the standard procedure of the Takara kit (RR820A, Takara). All data were analysed by the LightCycler^®^ 96 SW 1.1 software, the threshold period (Ct) was determined and the mRNA expression of the specific gene in the target sample was calculated by Ct values. Primer sequences for the specific genes used in this study are listed in Table 1.

**Table 1:** Primer Sequences for RT‐PCR

Gene	Forward primer	Reverse primer
MCP‐1	TAAAAACCTGGATCGGAACCAAA	GCATTAGCTTCAGATTTACGGGT
TNF‐α	GCGACGTGGAACTGGCAGAAG	GCCACAAGCAGGAATGAGAAGAGG
IL‐1β	TCGCAGCAGCACATCAACAAGAG	TGCTCATGTCCTCATCCTGGAAGG
ACTIN	AGCTGCTTCTGCGGCTCTAT	GTGGACAGTGAGGCCAGGAT

### 2.9 Statistics

Qualitative data including Western blots and various morphological images were representatives of at least three experiments. Quantitative data were expressed as means ± SD (standard deviation) and statistical analysis were performed using GraphPad Prism software. The 2‐tailed unpaired or paired Student’s *t* tests were used to determine the statistical difference between two groups. ANOVA followed by Tukey's post hoc tests were used to determine statistical differences between multiple groups. The value of *P* < 0.05 was considered significantly different.

## 3 RESULTS

### 3.1 NAM reduces UUO‐induced renal interstitial fibrosis

**Figure 1:**
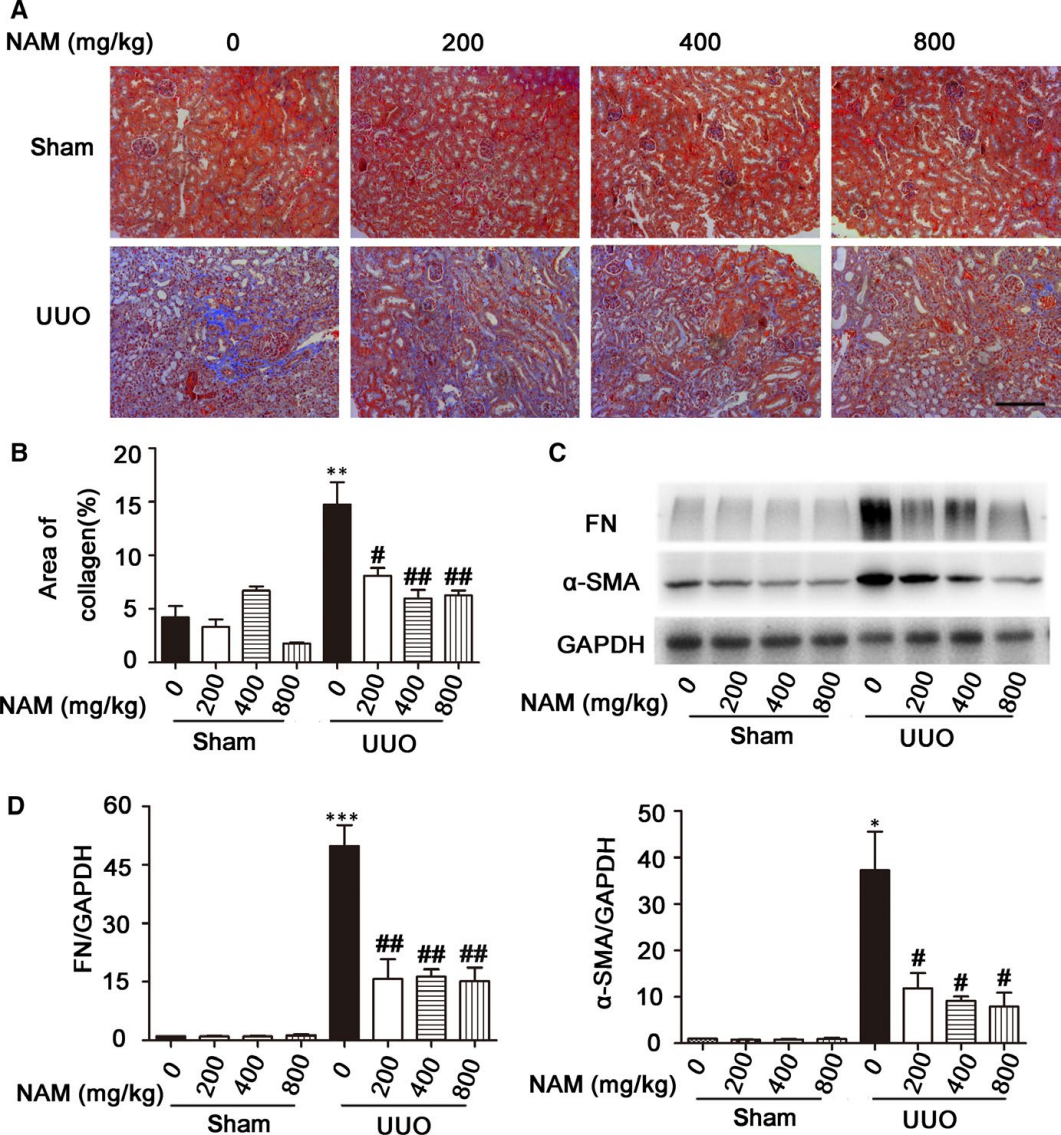
Nicotinamide (NAM) attenuates unilateral urethral obstruction (UUO)‐induced renal interstitial fibrosis. C57BL/6 mice were subjected to UUO surgery or sham operation. Different doses of NAM or saline were intraperitoneally injected an hour before the surgery and daily thereafter. The mice were sacrificed at 14 days after surgery to collect obstructed kidneys for histological analysis, Western blot and real‐time RT‐PCR analysis. (A) Representative images for Masson staining. Scale bar: 200 µmol/L. (B) Quantitative analysis of Masson staining. Data were expressed as mean ± SD (n = 5). **P < 0.01, significantly different from the sham group; ^#^ *P* < 0.05, ^##^ *P* < 0.01, significantly different from the UUO group. (C) Representative images of Western blot of FN (fibronectin), α‐SMA (α‐smooth muscle actin) and GAPDH (loading control). (D) Densitometric analysis of FN and α‐SMA. The ratio of FN/GAPDH or α‐SMA/GAPDH of sham control was arbitrarily set as 1, and the ratios of other groups were normalized with control to determine fold changes. These data were expressed as mean ± SD (n = 3). *P < 0.05, ***P < 0.001, significantly different from the sham group (with 0 NAM), ^#^ *P* < 0.05, ^##^ *P* < 0.01, significantly different from UUO (with 0 NAM)

We examined the effect of NAM on renal fibrosis during UUO. Different doses of NAM were given to mice with UUO. After 14 days, the mice were sacrificed and the obstructed kidney was taken for morphological and biochemical analysis. We first used Masson trichrome staining to detect collagen deposition in tissues of the obstructed kidneys. As shown in Figure 1A, sham control kidneys had minimal staining regardless of NAM. UUO induced significant increases in positive Masson staining (blue), which were notably reduced by 200‐800 mg/kg NAM. We further quantified the fibrosis staining by morphometric analyses using ImageProPlus. After two weeks of UUO, there was about 15% of renal tissue area stained positive, which was reduced to about 7% by NAM (Figure 1B). Furthermore, we examined the expression of fibrosis protein markers, including fibronectin (FN) and α‐smooth muscle actin (α‐SMA). The immunoblot results showed that both FN and α‐SMA accumulated dramatically in the obstructed kidney tissues, and this accumulation was significantly suppressed by the administration of NAM (Figure 1C). Semi‐quantification by densitometry further verified this conclusion (Figure 1D). Interestingly, the effect of NAM on renal fibrosis did not show a good dose‐dependence, suggesting the possibility of reaching the maximal effect at 200 mg/kg. These results suggest that NAM has inhibitory effects on renal interstitial fibrosis.

### 3.2 UUO‐induced tubule atrophy is alleviated by NAM

**Figure 2:**
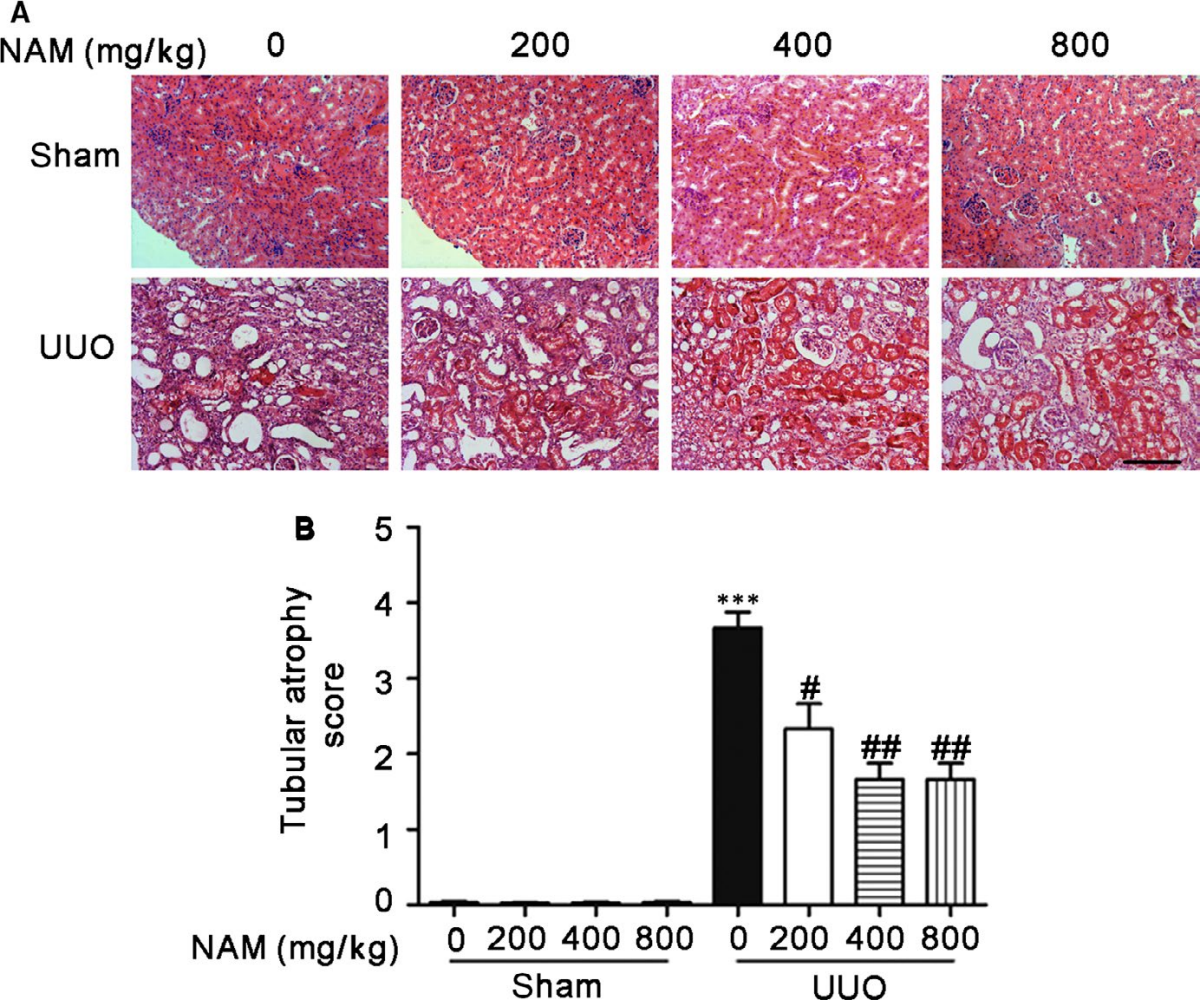
Nicotinamide (NAM) reduces unilateral urethral obstruction (UUO)‐induced tubular atrophy. C57BL/6 mice were subjected to UUO surgery or sham operation. Different doses of NAM or saline were intraperitoneally injected an hour before the surgery and daily thereafter. The mice were sacrificed at 14 days after surgery to collect obstructed kidneys for haematoxylin and eosin histological staining. (A) Representative images of haematoxylin and eosin staining. Scale bar: 200 µmol/L. (B) Tubular atrophy score of haematoxylin and eosin staining. Tubular atrophy was graded by 0, 1 (1%‐25%), 2 (26%‐50%), 3 (51%‐75%), 4 (76%‐100% tubules showing atrophy). These data were expressed as mean ± SD (n = 6). ***P < 0.001, significantly different from the sham group (with 0 NAM), ^#^ *P* < 0.05, ^##^ *P* < 0.01, significantly different from the UUO group (with 0 NAM)

Tubular atrophy, characterized by the stretching and thinning of kidney tubule cells with dilation of tubular lumen, often occurs during renal interstitial fibrosis.^3^, ^20^, ^21^ As a matter of fact, atrophic tubular cells may produce pro‐fibrotic factors for the initiation and progression of renal interstitial fibrosis.^2^, ^3^, ^22^, ^23^ To examine the effect of NAM on UUO‐induced tubular atrophy, we performed haematoxylin and eosin staining of obstructed kidney tissues. As shown in Figure 2, UUO caused significant renal damage, especially in the tubular structure, where many tubules were dilated and atrophic. The administration of NAM partially reduced the amount of atrophic tubules (Figure 2A). This result was confirmed by statistical analysis of tubular atrophy score (Figure 2B). Thus, NAM can help reduce UUO‐induced tubular atrophy.

### 3.3 NAM suppresses UUO‐induced tubular cell apoptosis

**Figure 3:**
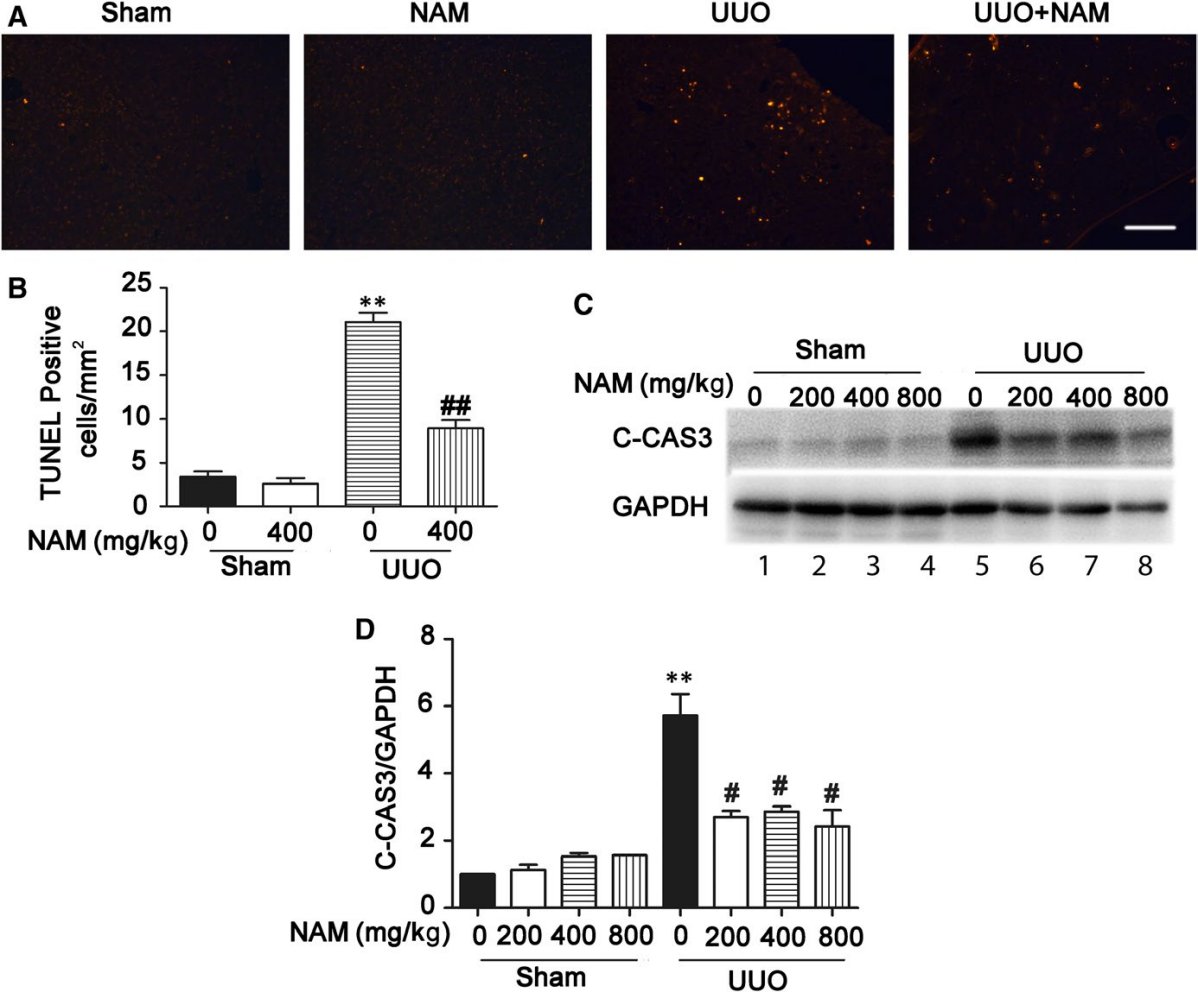
Nicotinamide (NAM) suppresses tubular cell apoptosis in unilateral urethral obstruction (UUO). C57BL/6 mice were subjected to UUO surgery or sham operation. Different doses of NAM or saline were intraperitoneally injected an hour before the surgery and daily thereafter. The mice were sacrificed at 14 days after surgery to collect obstructed kidneys for TUNEL staining. (A) Representative images of TUNEL staining. Scale bar: 200 µmol/L. (B) Quantitative analysis of TUNEL positive cells/mm^2^. Data were expressed as mean ± SD (n = 7), **P < 0.01, significantly different from the sham group with 0 NAM. ^##^ *P* < 0.01, significantly different from the UUO group with 0 NAM. (C) Representative images of Western blot of cleaved‐caspase3 (C‐CAS3). GAPDH was used as a loading control. (D) Densitometric analysis of cleaved‐caspase3 (C‐CAS3). The ratio of C‐CAS3/GAPDH of sham control was arbitrarily set as 1, and the ratios of other groups were normalized with the control to determine fold changes. These data were expressed as mean ± SD (n = 3). **P < 0.01, significantly different from the sham group with 0 NAM, ^#^ *P* < 0.05, significantly different from the UUO group with 0 NAM

Tubular apoptosis contributes significantly to the progressive cell loss and renal degeneration in renal interstitial fibrosis. It is also closely related to tubular atrophy and particularly its progression to tubular atresia.^2^, ^3^, ^23^ To determine the effect of NAM on tubular apoptosis in UUO, we first performed TUNEL (terminal deoxynucleotidyl transferase‐mediated dUTP nick end labelling) staining in kidney tissues. As shown in Figure 3A,B, while almost no TUNEL‐positive cells were detected in sham‐operated mice, about 20 apoptotic cells/mm^2^ kidney tissue were detected after 14 days of UUO. Importantly, administration of 400 mg/kg NAM reduced the number of apoptotic cells to 9. We further examined caspase activation by immunoblot analysis of active or cleaved caspase 3 (C‐CAS3). As shown in Figure 3C, UUO induced an obvious increase in cleaved caspase 3 (lane5 vs lane1), which was attenuated by 200‐800 mg/kg NAM (lane 6, 7, 8 vs lane 5). The blot result was further verified by densitometric analysis (Figure 3D). Together, these results indicate that NAM reduces apoptosis in UUO.

### 3.4 UUO‐induced renal inflammation is alleviated by NAM

**Figure 4:**
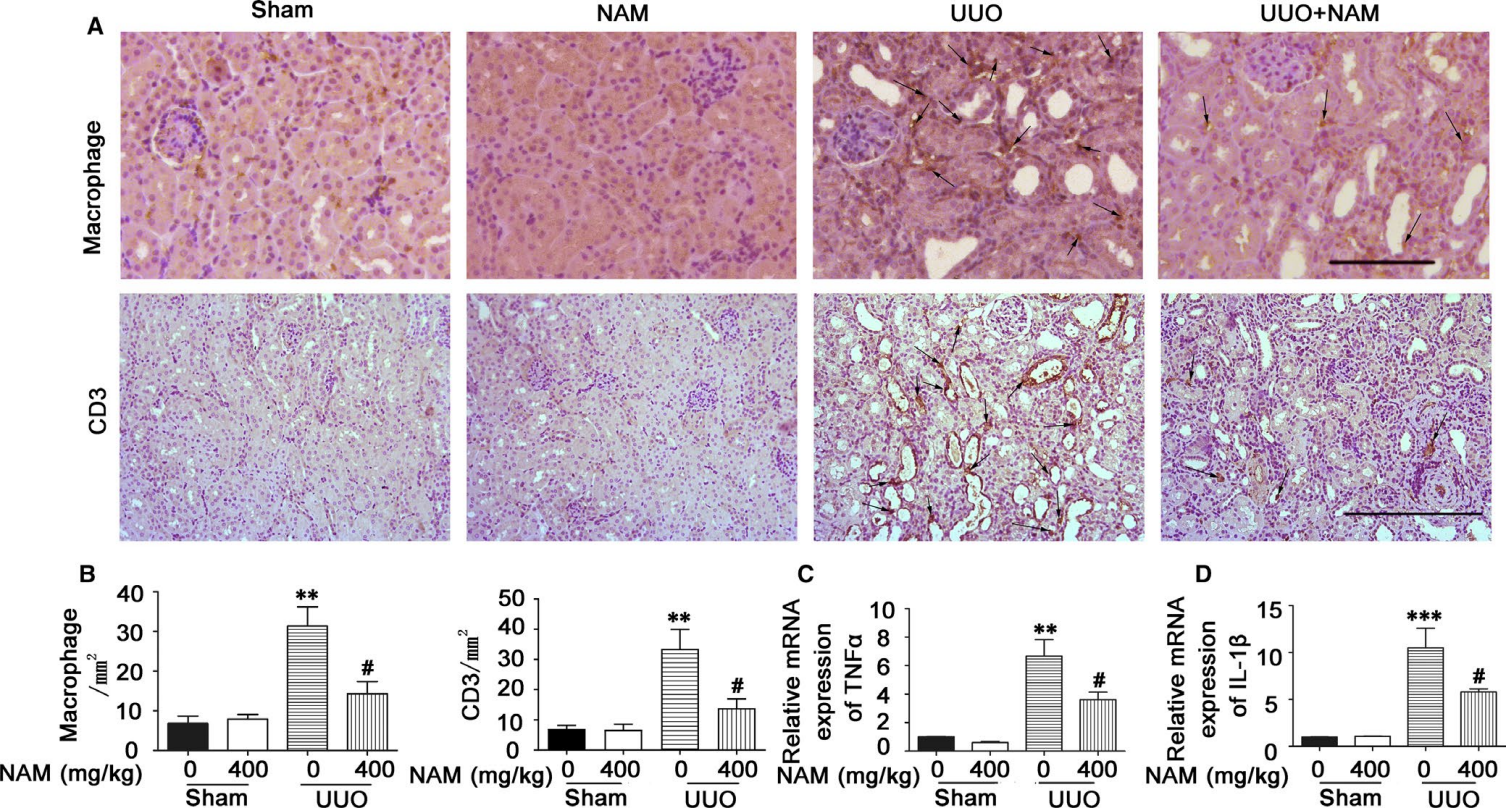
Unilateral urethral obstruction (UUO)‐induced kidney inflammation is alleviated by nicotinamide (NAM). C57BL/6 mice were subjected to UUO surgery or sham operation. Different doses of NAM or saline were intraperitoneally injected an hour before the surgery and daily thereafter. The mice were sacrificed at 14 days after surgery to collect obstructed kidneys for F4/80 or CD3 immunostaining, or RNA extraction for real‐time RT‐PCR analysis. (A) Representative images of F4/80 (macrophage) and CD3 staining. Arrows: typical staining. Scale bar: 200 µmol/L. (B) Quantitative analysis of F4/80‐macrophage and CD3 staining. Data were expressed as mean ± SD (n = 5). **, *P* < 0.01, different from the sham group with 0 NAM, ^##^ *P* < 0.01, significantly different from the UUO group with 0 NAM. (C) TNF‐α expression. Data were expressed as mean ± SD, **P < 0.01, different from the sham group with 0 NAM, ^#^ *P* < 0.05, significantly different from the UUO group with 0 NAM. (D) IL‐1β expression. Data are expressed as mean ± SD (n = 9), ***P < 0.001, different from the sham group with 0 NAM, ^#^ *P* < 0.05, significantly different from the UUO group with 0 NAM

Inflammation is another important factor for the initiation and progression of renal interstitial fibrosis diseases.^2^, ^3^, ^5^, ^24^ In this aspect, infiltration and phenotypic change of macrophages play a critical role.^25^, ^26^ To examine the role of NAM in UUO‐associated inflammation, we examined the infiltration of T cells and macrophages in kidney tissues. We further analysed the expression of pro‐inflammatory cytokines, including tumour necrosis factor alpha (TNF‐α) and interleukin‐1β (IL‐1β). As shown in Figure 4A, T cells (CD3 staining) and macrophages (F4/80 staining) were very rarely detected in sham‐control mice kidneys. Following UUO, both T cells and macrophages accumulated in kidney tissues, and notably, this accumulation was suppressed in the presence of NAM. Counting of the infiltrated cells further verified the effect of NAM (Figure 4B). Meanwhile, UUO induced TNF‐α and IL‐1β as shown by RT‐PCR analysis of mRNA expression, which was also suppressed by NAM (Figure 4C,D). These observations suggest that NAM has anti‐inflammatory function in UUO.

### 3.5 NAM attenuates TGF–β‐induced pro‐fibrotic changes in cultured proximal tubular cells

**Figure 5:**
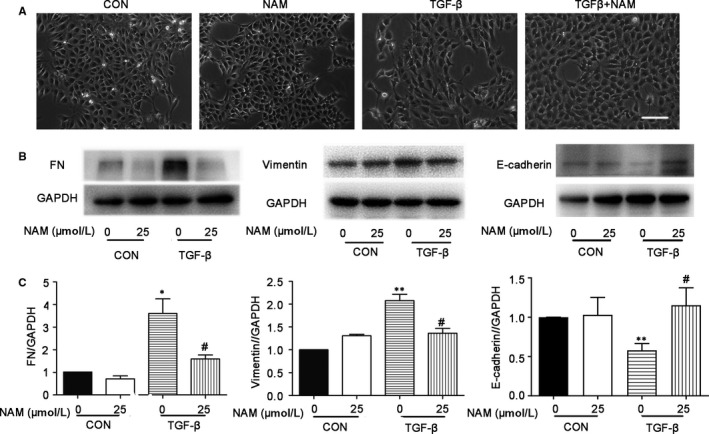
Nicotinamide (NAM) attenuates TGF–β‐induced pro‐fibrotic changes in BUMPT cells. BUMPT cells were untreated (control) or treated with 5ng/ml TGF‐β for 48 h in the absence or presence of 25 μmol/L NAM (NAM). After treatment, the cells were recorded for morphology, and lysate collected for immunoblot analysis. (A) Representative images of cell morphology under light microscope. Scale bar: 50 µmol/L. (B) Representative images of immunoblot analysis of fibronectin (FN), vimentin, E‐cadherin and GAPDH (protein loading control). (C) Densitometric analysis of FN, vimentin and E‐cadherin. The ratio of FN/GAPDH, vimentin/GAPDH or E‐cadherin/GAPDH of sham control was arbitrarily set as 1, and the ratios of other groups were normalized with the control to determine fold changes. These data were expressed as mean ± SD (n = 3). *P < 0.05, **P < 0.01, significantly different from the control group with 0 NAM, ^#^ *P* < 0.05, significantly different from the TGF‐β group with 0 NAM

TGF‐β is a key factor in renal interstitial fibrosis that may affect multiple cell types including renal proximal tubules.^28^, ^29^ As a result, TGF‐β treatment of renal tubular cells is a commonly used in vitro model for renal tubulointerstitial fibrosis research. Therefore, we determined the effect of NAM on TGF–β‐induced pro‐fibrotic changes in BUMPT cells, a mouse proximal tubular cell line. The cells were treated with 5 ng/mL TGF‐β with or without 25 μmol/L NAM for 48 hours. As shown in Figure 5, control BUMPT cells formed a typical cobblestone monolayer with intact cell‐cell connection. Upon TGF‐β treatment, the cells assumed a spindle‐shaped morphology with intercellular connection diminished. NAM, added together with TGF‐β, could partially prevent the morphological changes (Figure 5A). In Western blot, we detected the expression of fibronectin (FN, a fibrotic matrix protein) and Vimentin (a cell de‐differentiation marker) in TGF–β‐treated cells, whereas E‐cadherin was decreased (Figure 5B,C). Importantly, the changes of these proteins during TGF‐β treatment were largely attenuated by NAM (Figure 5B,C), further supporting the anti‐fibrotic effects of NAM on renal tubular cells.

### 3.6 NAM inhibits TGF–β‐induced apoptosis in proximal tubular cells

**Figure 6:**
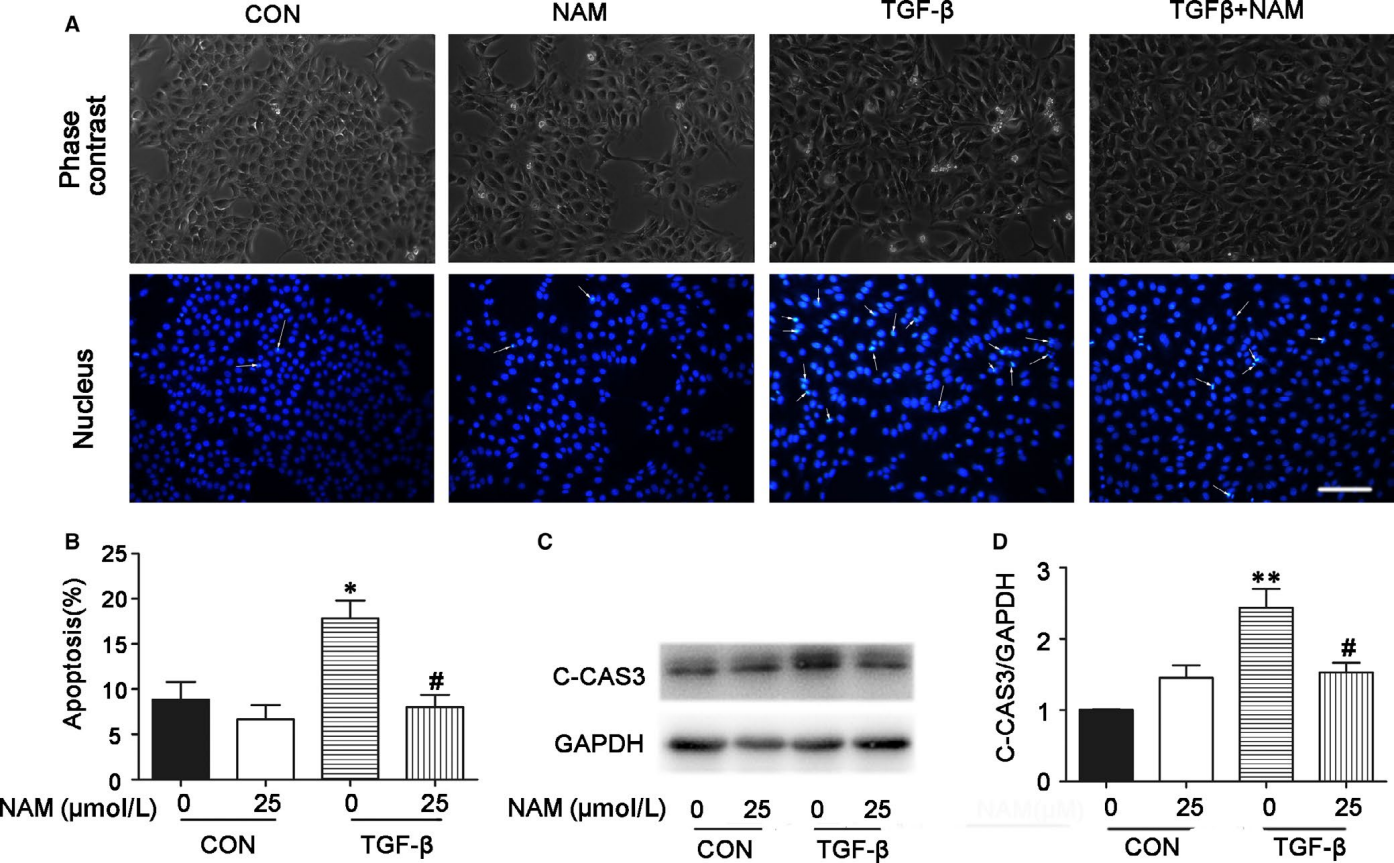
Nicotinamide (NAM) attenuates cell apoptosis during TGF‐β treatment of BUMPT cells. BUMPT cells were untreated (control) or treated with 5ng/ml TGF‐β for 48 h in the absence or presence of 25 μmol/L NAM (NAM). After treatment, the cells were stained with Hoechst33342 to record cellular and nuclear morphologies, or cell lysate collected for immunoblot analysis. (A) Representative images of cellular and nuclear morphologies. Apoptotic cells showed bright, condensed and/or fragmented cell bodies in phase contrast microscopy, while their nuclei were condense and sometimes fragmented in Hoechst33342 staining (arrows: typical apoptotic nuclei). Scale bar: 50µM. (B) Statistical analysis of apoptosis. These data were expressed as mean ± SD (n = 5). *P < 0.05, different from the control group with 0 NAM, ^#^ *P* < 0.05, significantly different from the TGF‐β group with 0 NAM. (C) Representative image of Western blot of cleaved‐caspase3 (C‐CAS3), GAPDH was used as an internal loading control. (D) Densitometric analysis of cleaved‐caspase3 (C‐CAS3) signals. The C‐CAS3/GAPDH ratio of control was arbitrarily set as 1, and the signals of other groups were normalized with the control to determine fold changes. These data were expressed as mean ± SD (n = 4). **P < 0.01, significantly different from the control group with 0 NAM, ^#^ *P* < 0.05, significantly different from the TGF‐β group with 0 NAM

We showed the inhibitory effect of NAM on renal tubular apoptosis during UUO in mice (Figure 3). In vitro, TGF‐β treatment induced a marginal level of apoptosis in BUMPT cells. We recorded cellular and nuclear morphologies following Hoechst staining, which revealed apoptotic cells with cellular and nuclear condensation and fragmentation (Figure 6A). In counting, there were about 20% apoptotic cells after TGF‐β treatment for 48 hours. Interestingly, the addition of NAM significantly decreased apoptosis during TGF‐β treatment (Figure 6A,B). This morphological finding was verified by Western blot analysis of cleaved caspase 3. As shown in Figure 6C,D, TGF‐β increased the expression of cleaved caspase3, which was reduced by 25 μmol/L NAM. Thus, both in vivo and in vitro data support the conclusion that NAM reduces renal tubular apoptosis in renal fibrosis.

### 3.7 Inflammatory response of BUMPT cells induced by TGF‐β is inhibited by NAM

**Figure 7:**
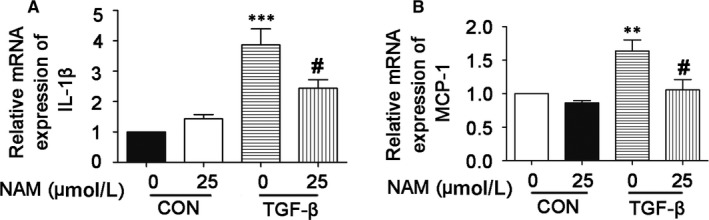
Inflammatory response of BUMPT cells induced by TGF‐β is inhibited by nicotinamide (NAM). BUMPT cells were untreated (control) or treated with 5 ng/mL TGF‐β for 48 h in the absence or presence of 25 μmol/L NAM (NAM). After treatment, RNA was extracted for real‐time RT‐PCR analysis of the expression of IL‐1β and MCP‐1. (A) IL‐1β expression. Data were expressed as mean ± SD (n = 9). ***P < 0.001, different from the control group with 0 NAM, ^#^ *P* < 0.01, significantly different from the TGF‐β group with 0 NAM. (B) MCP‐1 expression. Data are expressed with mean ± SD. **P < 0.01, different from the control group with 0 NAM, ^#^ *P* < 0.05, significantly different from the TGF‐β group with 0 NAM

The inflammatory response of renal tubular epithelial cells is a key point to the development of renal interstitial fibrosis.^2^, ^3^, ^5^, ^23^ To determine the effect of NAM on the inflammatory response of renal tubular cells, we analysed the expression of monocyte chemoattractant protein 1 (MCP‐1) and interlukin‐1 beta (IL‐1β). BUMPT cells were treated with TGF‐β with or without 25 μmol/L NAM for 48 hours to extract RNA for real‐time PCR analysis. As shown in Figure 7A,B, TGF‐β induced significantly increases in MCP‐1 and IL‐1 mRNA expression, which was partially yet significantly reduced by NAM. The results indicate that NAM may directly suppress the pro‐inflammatory response in renal tubular cells during renal interstitial fibrosis.

## 4 DISCUSSION

Renal interstitial fibrosis is a pathological hallmark of progressive kidney diseases in almost all etiologies. At present, there is an urgent need to identify effective treatments for renal fibrosis, which is generally considered to be irreversible. NAM is a member of the vitamin B family that was recently shown to have a therapeutic potential for AKI.^13^, ^14^ However, it is unknown whether NAM and other NAD^+^‐related compounds may have beneficial effects on chronic kidney pathologies, especially renal fibrosis. In this study, we have tested the effects of NAM in the in vivo model of UUO and in vitro model of TGFβ1‐treated proximal tubular cells. Our results demonstrate significant anti‐fibrotic activities of NAM in both models. Mechanistically, NAM may suppress renal tubulointerstitial fibrosis by alleviating tubular injury and related inflammation.

UUO in mice is a commonly used animal model of chronic kidney pathologies, including interstitial fibrosis.^30^, ^31^ In our study, NAM significantly reduced renal interstitial fibrosis during UUO in mice (Figure 1). This was shown by Masson trichrome staining of collagen fibrils or deposition. Moreover, NAM decreased the expression of pro‐fibrotic proteins, such as FN and α‐SMA. Renal interstitial fibrosis involves the crosstalk or interplay between multiple kidney cell types, among which renal tubular epithelial cells play an important role.^2^, ^3^, ^23^, ^33^ Consistently, we verified that NAM may suppress TGF–β‐induced fibrotic changes in cultured renal proximal tubular cells (Figure 5). Of note, the effect of NAM on TGF–β‐induced morphological changes in these cells was marginal, but its inhibitory effect on fibrosis protein (eg FN and α‐SMA) expression was evident. Together, these in vivo and in vitro results demonstrate the first evidence of the anti‐fibrotic effect of NAM in kidneys.

How does NAM inhibit renal interstitial fibrosis? With this question, we examined the effects of NAM on tubular injury and inflammation. During the development of renal interstitial fibrosis, tubular injury is characterized by tubular degeneration, including tubular cell death as well as tubular atrophy and atresia.^2^, ^3^, ^23^, ^33^ In our experiments, UUO induced atrophic tubules in mouse kidneys that was apparently reduced by NAM (Figure 2). In addition, NAM significantly reduced renal apoptosis during UUO, as shown by TUNEL assay and immunoblot analysis of active/cleaved caspase 3 (Figure 3). Moreover, TGF–β‐induced apoptosis in cultured proximal tubular cells was also decreased in NAM (Figure 6). These observations suggest that NAM may antagonize renal fibrosis by reducing tubular injury and/degeneration, including tubular atrophy and apoptosis.

Chronic or persistent inflammation is another important factor driving the progression of renal fibrosis.^2^, ^3^, ^5^, ^24^, ^25^ In our study, NAM showed significant anti‐inflammation effects. We specifically examined macrophage and T cell infiltration in renal tissues during UUO, which was significantly suppressed by NAM. In addition, NAM reduced the expression of pro‐inflammatory cytokines, such as TNF‐α and IL‐1β (Figure 3). Interestingly, cultured tubular cells produced pro‐inflammatory cytokines in response to TGF‐β1 treatment, and this production was also suppressed by NAM (Figure 7). These results, together, suggest that NAM may antagonize renal interstitial fibrosis by attenuating inflammation.

While our study demonstrates the effects of NAM on both tubular injury and inflammation, we postulate its primary or proximal action on renal tubular cells. In UUO, urine flow is blocked, resulting in the backup of urine in the obstructed kidney, which leads to tubular injury and cell death, inflammation and disruption of renal haemodynamics for fibrogenesis.^34^ Under this and other related conditions, tubular changes are considered the driving force for the development of renal interstitial fibrosis. It is known that injured or regenerating tubular cells may change to a secretory phenotype for the production and release of pro‐fibrotic factors that not only stimulate the activation and expansion of interstitial resident fibroblasts but also trigger inflammation. As such, amelioration of tubular injury or stress may have enormous effects on the occurrence and progression of renal interstitial fibrosis. The recent studies by Parikh and colleagues have demonstrated a critical role of NAD biosynthesis in maintaining renal tubular cell viability and function in AKI in both mice and humans.^13^, ^14^ In cells, NAM is a key precursor for NAD biosynthesis. Our current study indicates that supplementation of NAM can reduce UUO‐associated tubular atrophy and apoptosis, further supporting the role of NAM‐mediated NAD biosynthesis in cellular homeostasis and viability of renal tubules. By reducing tubular injury and stress, NAM may suppress inflammation and interstitial fibroblast activation.

It remains unclear how NAM protects renal tubular cells. As mentioned above, NAM is a key precursor for the biosynthesis of NAD+, a critical coenzyme and electron acceptor in glycolysis and the Krebs cycle. It is therefore possible that NAM may help the cells maintain their bioenergetics status for survival during injury and stress. In addition, NAD + is a substrate for several non‐redox enzymes including poly ADP‐ribose polymerase (PARP) and sirtuins. Interestingly, these enzymes have been implicated in kidney injury. For example, PARP‐1 has been reported to inhibit glycolysis during ischaemic kidney injury by poly(ADP‐ribosyl)ation of the key glycolytic enzyme glyceraldehyde‐3‐phosphate dehydrogenase (GAPDH)^35^ and inhibition of PARP‐1 can protect against ischaemic kidney injury by preserving tubular cell ATP.^36^ NAM has been reported to have protective effects in a rat model of Alzheimer's disease by inhibiting PARP‐1.^37^ Sirtuins are a family of NAD‐dependent deacetylases that play regulatory functions in a variety of tissues and organs, including kidneys.^38^ Of much relevance to our current study, NAM was recently shown to reduce ageing‐associated susceptibility to AKI in a Sirt1‐dependent manner.^39^ Future studies should investigate the effects of NAM on cellular bioenergentics, PARP‐1 and sirtuins in experimental models of renal fibrosis to gain better understanding of its anti‐fibrotic effect.

## CONFLICT OF INTEREST

The authors declare no competing financial interests in relation to the work described in this study.

## AUTHORS’ CONTRIBUTIONS

Zheng Dong, Juan Cai, Meiling Zheng conceived the study; Meiling Zheng performed the experiments; Meiling Zheng, Juan Cai, Zhiwen Liu, Shaoqun Shu, Ying Wang, Chengyuan Tang, Zheng Dong analysed the results and approved the publication; Meiling Zheng prepared the first draft; Zheng Dong, Chengyuan Tang, Juan Cai revised the paper.
